# Identification of a novel autophagy-related prognostic signature and small molecule drugs for glioblastoma by bioinformatics

**DOI:** 10.1186/s12920-022-01261-5

**Published:** 2022-05-12

**Authors:** Dongjiao Wang, Yuxue Jiang, Tie Wang, Zhe Wang, Fei Zou

**Affiliations:** 1 Department of Gynecological Oncology, The First Hospital of Jilin University, Changchun, 130021 Jilin China; 2 Department of Public Laboratory Platform, The First Hospital of Jilin University, Changchun, 130061 Jilin China; 3 Department of Laboratory, The First Hospital of Jilin University, Changchun, 130021 Jilin China; 4 Department of Gastrointestinal Oncology Cancer Hospital of China Medical University, Liaoning Cancer Hospital and Institute, Shenyang, 110042 China; 5 Department of Pediatrics, The First Hospital of Jilin University, Changchun, 130021 Jilin China

**Keywords:** Autophagy, Gene signature, Prognosis, Glioblastoma, Mutation, LASSO cox regression analysis

## Abstract

**Objective:**

To explore the autophagy-related prognostic signature (ARPs) via data mining in gene expression profiles for glioblastoma (GBM).

**Methods:**

Using the Cancer Genome Atlas (TCGA) database, we obtained 156 GBM samples and 5 adjacent normal samples, and denoted them as discovery cohort. Univariate Cox regression analysis was used to screen autophagy genes that related to GBM prognosis. Then, the least absolute shrinkage and selection operator Cox regression model was used to construct an autophagy-based ARPs, which was validated in an external cohort containing 80 GBM samples. The patients in the above-mentioned cohorts were divided into low-risk group and high-risk group according to the median prognostic risk score, and the diagnostic performance of the model was assessed by receiver operating characteristic curve analyses. The gene ontology and Kyoto encyclopedia of genes and genomes pathway enrichment analyses were performed between the high-risk and low-risk patients. Additionally, the genetic features of ARPs, such as genetic variation profiles, correlations with tumor-infiltrating lymphocytes (TILs), and potential drug sensitivity, were further assessed in the TCGA-GBM data set.

**Results:**

A signature of ARPs including *NDUFB9, BAK1, SUPT3H, GAPDH, CDKN1B, CHMP6,* and *EGFR* were detected and validated. We identified a autophagy-related prognosis 7-gene signature correlated survival prognosis, immune infiltration, level of cytokines, and cytokine receptor in tumor microenvironment. Furthermore, the signature was tested in several pathways related to disorders of tumor microenvironment, as well as cancer-related pathways. Additionally, a range of small molecular drugs, shown to have a potential therapeutic effect on GBM.

**Conclusions:**

We constructed an autophagy-based 7-gene signature, which could serve as an independent prognostic indicator for cases of GBM and sheds light on the role of autophagy as a potential therapeutic target in GBM.

**Supplementary Information:**

The online version contains supplementary material available at 10.1186/s12920-022-01261-5.

## Background

Glioblastoma is known as the most frequent primary malignant tumor of the brain, accompanied with 15–23 months for the OS, and < 6% for the 5-year survival rate [1, 2]. Surgery is currently the first standard option for GBM, followed by adjuvant radiation therapy and chemotherapy, and the prognosis of GBM patients remains poor [3]. So it is urgent to identify effective markers for prognostic prediction and predictors for clinical therapeutic response. Over the past decade, histologic grade, tumor stage, and residual tumor size have been revealed as prognostic factors for GBM patients. Moreover, studies conducted at the molecular level revealed the isocitrate dehydrogenase (*IDH*) mutation status, methylation of the O-6-methylguanine-DNA methyltransferase (*MGMT*) gene promoter, and 1p/19q co-deletion are also prognostic markers of GBM [4–6]. Despite advances in molecular-level prediction, the overall outcome of GBM patients remains undesirable.

As a mechanism for maintaining cellular homeostasis, autophagy is an intracellular lysosomal degradation process [7–9], including cancer initiation and development. Hundreds of scholars have concentrated on the presentation of an optimum model for autophagy-related prognostic signature [10, 11], but the GBM-specific genetic profile has not been identified yet, and early markers for prognosis of GBM are still unknown. In recent years, the crucial roles of autophagy have been reported in a variety of diseases [12, 13]. It has also been demonstrated that there is a close correlation between autophagy dysfunction and GBM. For instance, Vehlow et al. emphasized the role of discoidin domain receptor tyrosine kinase 1 (*DDR1*) in sensitization of GBM cells to combination therapies via inducing autophagic cell death [14]. Recently, Temozolomide (TMZ) has been introduced to inhibit tumor growth via the induction of autophagy [15]. Autophagy is taken as a potential therapeutic target into consideration for both cancer prevention and therapy, whereas its mechanisms and role require further clarification. The present research was conducted for further systemic assessment of the prognostic value of these autophagy-related genes (ARGs) in GBM.

In the current study, we detected an ARPs in GBM samples from the TCGA and gene expression omnibus (GEO) data sets. This ARPs can be used as an independent predictor with the prognostic value for patients with GBM. In addition, we analyzed the genetic alteration and expression characteristics of the signature genes, the relationship with tumor microenvironments, and predicting novel potential small molecule drug candidates for GBM. The results of this study clarify the important role of ARPs in GBM. Taken together, our study demonstrates that autophagy plays an essential role in the progression, and prognosis in GBM, and suggests an autophagy-related signature as a promising prognostic biomarker and potential therapeutic target for GBM patients.

## Materials and methods

### Screening of autophagy-related gene sets

The autophagy-related gene sets were retrieved from the Human Autophagy Database that had been reported in the literature as being involved in the autophagy process (HADb: http://www.autophagy.lu/index.html) [16] and hallmark gene sets of Molecular Signatures Database v5.2. gene sets [17] (https://www.gsea-msigdb.org/gsea/msigdb). We collected 531 ARGs after removing the duplications (Additional file 1).

### Samples and data collection

High-throughput RNA sequencing (RNA-seq) data involved 156 tumor samples and 5 normal samples, and corresponding clinicopathological parameters of patients with GBM were obtained from TCGA (https://tcga-data.nci.nih.gov/tcga/), and served as the discovery cohort (TCGA-GBM) to develop an autophagy-related GBM prognostic signature. The TCGA data set was normalized using the FPKM method [18]. In addition, the GSE7696 data set was used as an independent external validation cohort containing 80 GBM samples.

Four independent GBM microarray cohorts synthesized by the GPL570 platform extracted from GEO database (accession number: GSE4290, GSE15824, GSE7696, GSE50161) [19–21] were integrated. The Robust Multi-array Average method was used to normalize raw microarray datasets [22]. The ComBat method was used to remove any batch effects [23]. Finally, a total of 207 tumor samples and 42 normal samples were acquired and used for principal components analysis (PCA). Finally, all the data analyzed in our research were presented in the Additional file 4.

The cBioPortal (http://www.cbioportal.org) is a repository of cancer genomics datasets [24]. Mutation data for signature genes were downloaded from the cBioPortal.

### Statistical analysis

First, univariate Cox regression analysis was undertaken to detect the autophagy-related genes related to GBM prognosis. P-value < 0.05 was set as the cutoff value, and a total of 30 ARGs were found as candidate genes (Additional file 2). Second, the 30 significant prognostic genes were further chosen by LASSO analysis, which could effectively prevent over fittings, address the multicollinearity problem. Subsequently, we acquired 19 prognosis-related autophagy genes (Additional file 3). Then, a multivariate Cox proportional hazards regression model was established using the 19 candidate prognostic autophagy genes. To develop an explanatory, predictive model, the values of *Akaike information criterions* (AICs) were computed for all candidate models, and finally, an optimal model with the minimal AIC was selected [25]. By weighting the multivariate Cox proportional hazards regression coefficients, a 7-gene risk signature was constructed [26], and a risk score model of the autophagy-related genes prognostic makers was established according to the following formula:

$$\begin{aligned} {\text{Prognostic}}\;{\text{risk}}\;{\text{score}} & = \left( { - 0.379 \times {\text{expression}}\;{\text{level}}\;{\text{of}}\;NDUFB9} \right) + \left( { - 0.867 \times {\text{expression}}\;{\text{level}}\;{\text{of}}\;BAK1} \right) \\ & \quad + \left( { - 0.529 \times {\text{expression}}\;{\text{level}}\;{\text{of}}\;SUPT3H} \right) + \left( {0.462 \times {\text{expression}}\;{\text{level}}\;{\text{of}}\;GAPDH} \right) \\ & \quad {\text{ + }}\left( { - 0.694 \times {\text{expression}}\;{\text{level}}\;{\text{of}}\;CDKN1B} \right) + \left( {1.066 \times {\text{expression}}\;{\text{level}}\;{\text{of}}\;CHMP6} \right) \\ & \quad {\text{ + }}\left( { - 0.135 \times {\text{expression}}\;{\text{level}}\;{\text{of}}\;EGFR} \right). \\ \end{aligned}$$

Then, the prognostic risk score of each GBM patient was calculated, the patients were divided into high-risk and low-risk groups according to the median value of the risk score. Finally, to assess the prediction of GBM patients’ OS by the prognostic risk score model, the Kaplan–Meier (KM) method and log-rank test were used to compare survival curves between groups. A two-sided *p*-value of < 0.05 was considered statistically significant. Independent prognostic factors hazard ratios (HR) with 95% confidence intervals (95% CIs) were identified using the multivariate Cox proportional hazards regression model. The prediction accuracy of this ARPs was determined by ROC curves and area under the ROC curve (AUC) analyse, and the APRs was further validated by fitting the validation data set (GSE7696 cohort).

### GO and KEGG pathway analyses

To explore the potential molecular mechanisms underlying the ARPs in GBM, the KEGG and GO analyses of DEGs between the low-risk and high-risk groups were performed using DAVID software (https://david-d.ncifcrf.gov/) [27], and plotted in R using ggplot2. We considered terms significantly enriched with both the p- and q-values were < 0.05.

### Independent genetic alteration and expression characteristics of the signature genes

To test the independent genetic alteration and expression characteristics of the signature genes, we conducted analyses as follows:

First, the open-source cBioPortal database (http://www.cbioportal.org) was used for exploring cancer genetic alterations of the 7 signature genes with data retrieved from the TCGA-GBM database [24]. Second, the TISIDB database was used to analyze the interaction between signature genes and cytokine receptor expression. The TISIDB database (http://cis.hku.hk/TISIDB) is a web portal to analyze tumor and immune microenvironment interaction (such as TIICs, immunomodulators, and chemokines, etc.) [28]. The 7 signature genes which the strongest association with cytokines were selected for further analysis. Third, Tumor Immune Estimation Resource (TIMER) database was used to evaluate the abundance of TIICs from gene expression profiles [29]. TIMER is an online database (https://cistrome.shinyapps.io/timer/), which includes 10,897 samples across 32 cancer types and can provide a systematic analysis of immune infiltrates levels [30], using a deconvolution method.

### Screening of small molecule drugs by genomics of drug sensitivity in cancer database

Genomics of Drug Sensitivity in Cancer (GDSC) is a web-based platform for efficient parallelization of genomic analyses, including gene expression, methylation, single nucleotide variation, pathway activity, and drug targets analyses [31]. Here, GDSC was applied to analyze the drug sensitivity of the signature genes and evaluated the correlation between gene expression and half-maximal [50%] inhibitory concentration (IC50) of potential small molecule inhibitors. P-value < 0.05 was considered statistically significant.

## Results

### ARGs expression profiles showed distinct expression patterns

**Fig. 1:**
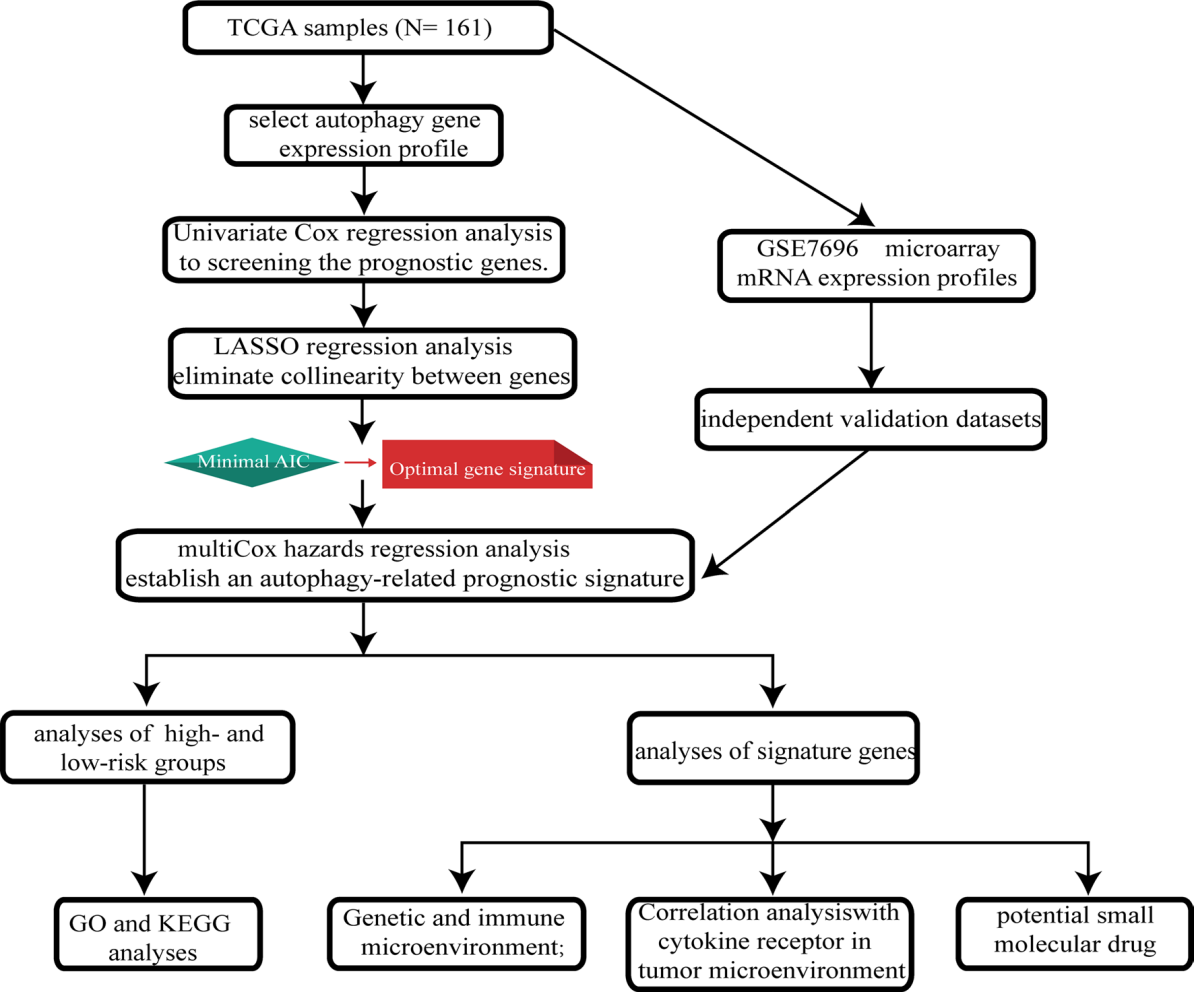
The flow diagram of this study

The study design is presented in Fig. 1. To detect the expression patterns of ARGs expression profile, a total of 207 GBM samples and 42 normal samples with ARGs expression data were retrieved from four GEO datasets. A principal components analysis comparing GBM tissues with normal tissues based on their expression profiles showed two significantly different distribution patterns (Additional file 5: Fig. S1). Normal samples were mainly distributed on the left side, while GBM samples were distributed on the right side (principal components (PC) 2, and PC1), suggesting distinctly different regulatory roles for the autophagy biological process in normal brain tissues and GBM tissues.

### ARPs construction and its association with GBM prognosis

**Fig. 2:**
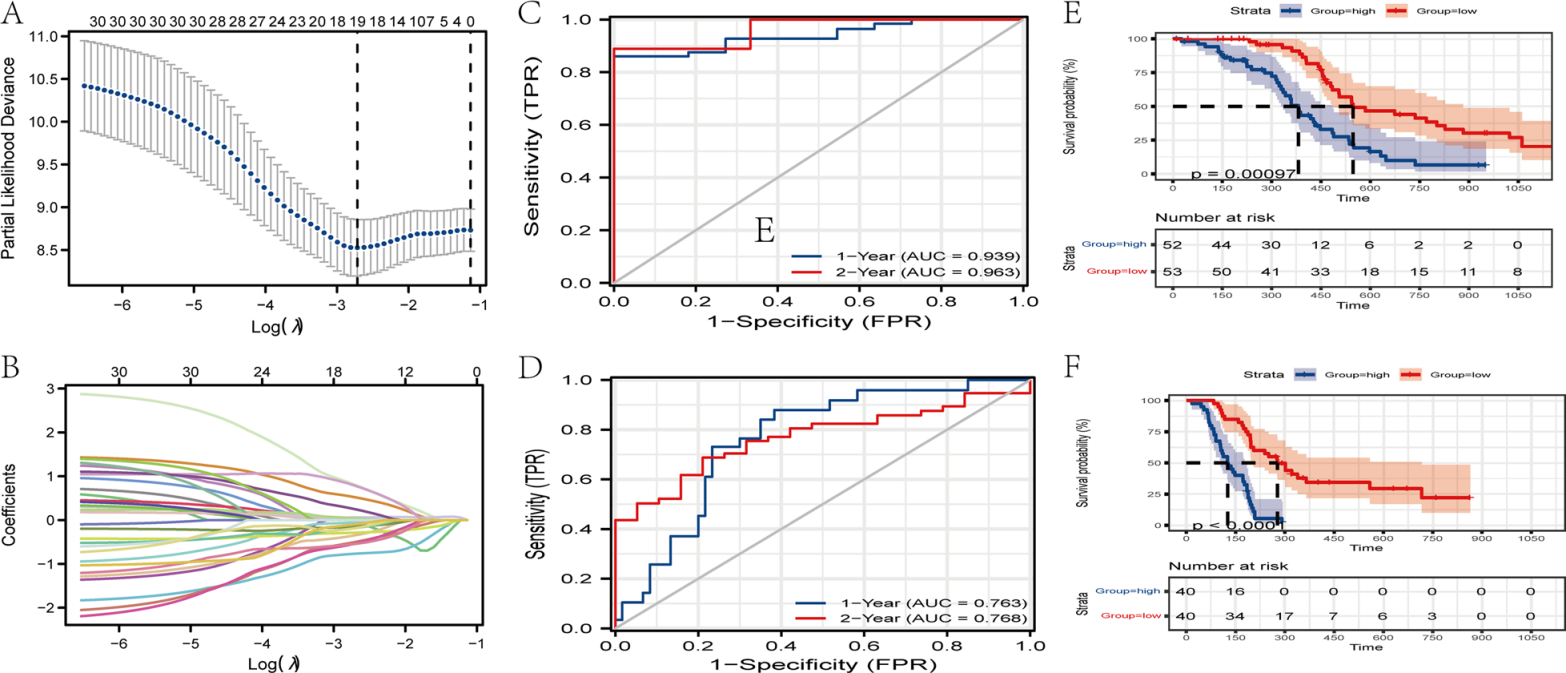
Using autophagy regulators to establish risk signature ARPs in GBM. These regulators were used to create risk signatures in TCGA and GEO datasets. **A** Parameter selection for tuning by tenfold cross-validation in the LASSO model. **B** LASSO coefficient profiles of the fractions of autophagy genes. **C**, **D** ARPs measured by the time-dependent receiver–operating characteristic (ROC) curves in the training cohort (TCGA), and validation cohort (GEO). **E**, **F** KM-curve for patients with high and low ARPs in the training cohort, and validation cohort

To further assess the role of ARGs in the prognosis of GBM patients, we investigated the relationship between autophagy-related genes and survival in GBM patients, and identified an ARPs that might be helpful in predicting the prognosis of GBM patients. First, we submitted autophagy-related genes to univariate Cox regression analysis, with a threshold value of *p* < 0.05, and, 30 promising candidates (Additional file 2) were identified. Subsequently, the LASSO Cox regression model was applied to overcome over-fitting and used to identify the most robust markers for prognosis, with the optimal λ value (lambda.min) of 0.0661 selected (Fig. 2A, B). Consequently, we identified a 7-gene signature that was significantly correlated with OS in GBM patients. For each GBM patient, a signature risk score was calculated via the prognostic risk score (see method). Patients were then divided into high/low-risk groups via the median risk score. Among the 7 genes, *GAPDH*, and *CHMP6* were identified as risk factors (HR > 1), while the other 5 genes (*NDUFB9, BAK1, SUPT3H, CDKN1B, EGFR*) were identified as protective factors (HR < 1) (Table 1).

**Table 1:** Multivariate Cox analysis among ARPs in the discovery cohort

Gene	Coef	HR	HR.95L	HR.95H	*p* Value
*NDUFB9*	− 0.3793	0.6843	0.4076	1.1486	0.1511
*BAK1*	− 0.8674	0.42	0.1989	0.8868	0.0229
*SUPT3H*	− 0.5297	0.5887	0.366	0.947	0.0289
*GAPDH*	0.4620	1.5873	1.0194	2.4714	0.0408
*CDKN1B*	− 0.6944	0.4993	0.2563	0.9727	0.0412
*CHMP6*	1.0663	2.9047	1.197	7.0485	0.0183
EGFR	− 0.1352	0.8735	0.7723	0.9879	0.0312

*Coef* coefficients, *HR* hazard ratios

To verify the ability of the 7-gene signature in predicting the prognosis of GBM patients, time-dependent ROC curve analysis was performed in the discovery cohort, and the area under the ROC curve was calculated (AUC: 0.939 at 1 year, 0.963 at 2 years) (Fig. 2C). Furthermore, KM survival curves showed that OS in the high-risk group was significantly poorer compared with that in the low-risk group in the TCGA cohort (p = 0.00097) (Fig. 2E). These results highlighted that the ARPs model possessed a relatively satisfactory predictive performance.

To validate the accuracy of the 7-gene signature, we used an independent set of GBM patients with OS and gene expressions information (GSE7696). ROC curve analyses demonstrated that this ARPs was capable of predicting OS in patients with GBM (AUC: 0.763 at 1 year, 0.768 at 2 years,) (Fig. 2D). Kaplan–Meier plots indicated significant differences between 5-year OS (p < 0.0001, Fig. 2F). Taking together, these results confirmed that the ARPs might be a better prediction tool, which might help clinical management of high-risk GBM patients.

### Pathway analyses in ARPs-risk grouping

**Fig. 3:**
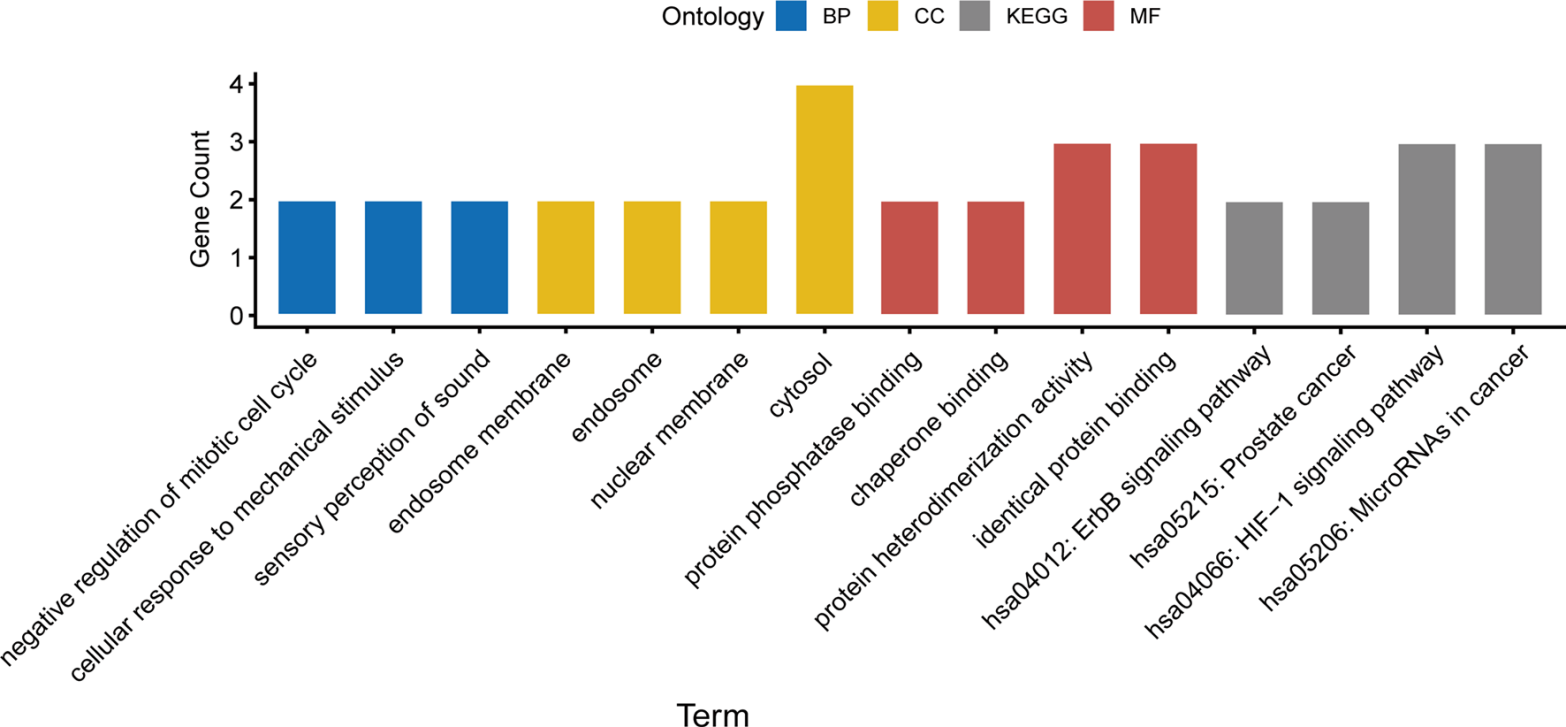
GO and KEGG pathway analyses of differentially expressed genes between high- and low-risk groups in TCGA data set

To investigate the underlying pathways behind the 7-gene signature, GO analysis, and significantly enriched KEGG pathways were found in the TCGA cohort. The top 11 GO terms are displayed in Fig. 3. In particular, negative regulation of the mitotic cell cycle, protein phosphatase binding, and identical protein binding were significantly enriched in the majority of the GO terms. KEGG analysis reveals that a great majority of the enriched pathways were frequently dysregulated pathways in cancer, such as HIF-1 signaling pathway, ErbB signaling pathway, and MicroRNAs in cancer (Fig. 3).

### Genetic and immune microenvironment characteristics of the seven signature genes

**Fig. 4:**
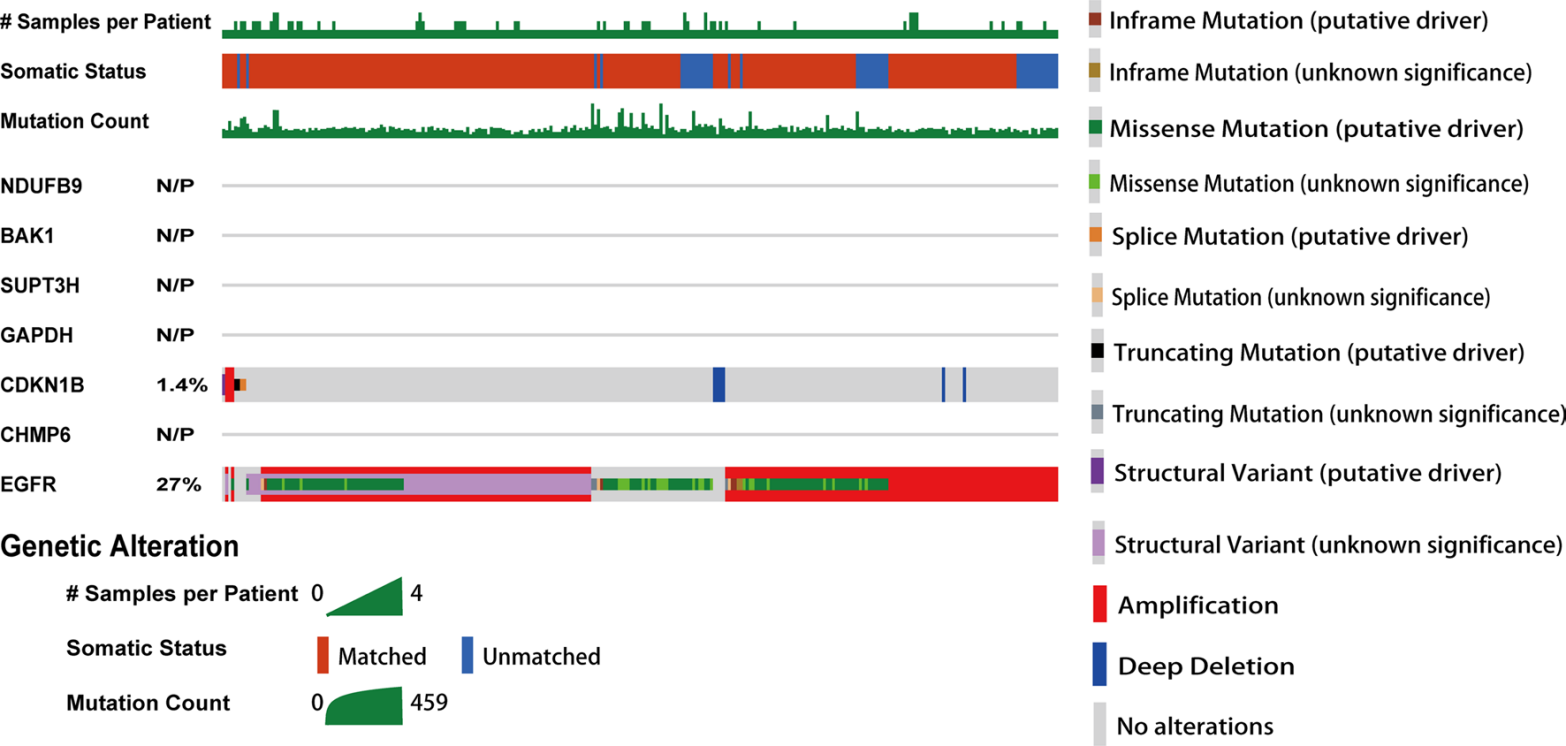
Genetic alterations of ARPs in GBM determined with the cBioPortal database

To explore the genetic features of the 7-gene signature, we analyzed the genetic variation profiles in 1004 cases that were retrieved from Glioblastoma (MSKCC, Clin Cancer Res 2019) using the cBioPortal database. We found that the signature genes exhibited no mutation or a low mutation frequency (Fig. 4). CDKN1B was represented with mutation frequencies of 1.4%. One particular gene EGFR, 27% of the total patients harbored the EGFR amplification and missense mutation.

**Fig. 5:**
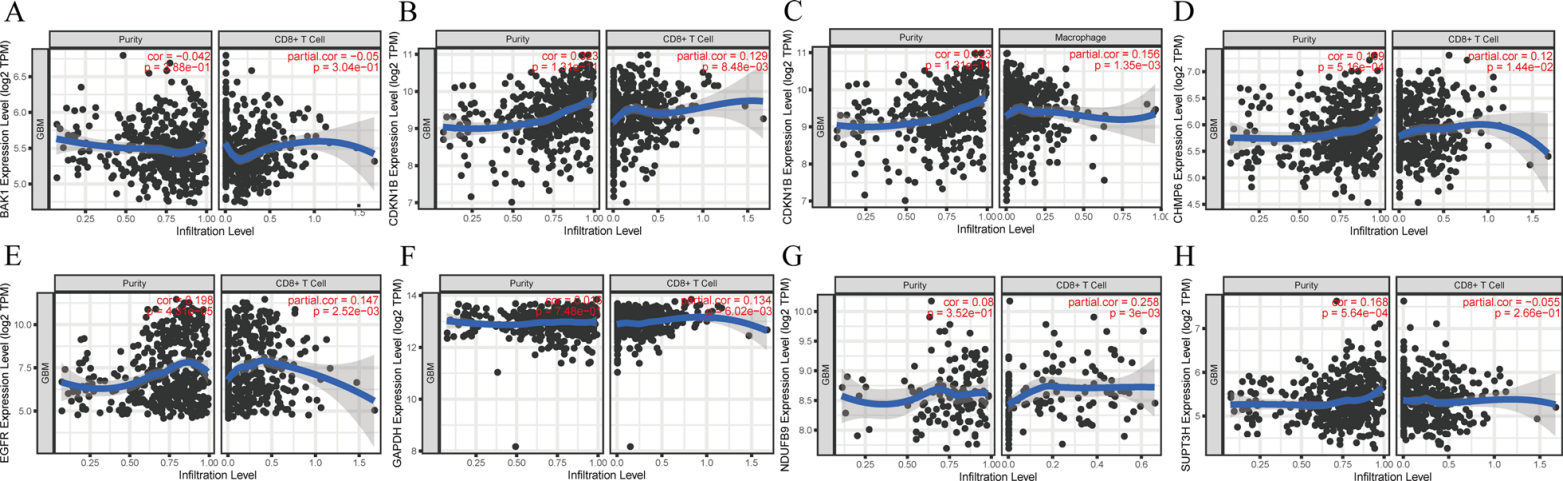
TIMER (https://cistrome.shinyapps.io/timer/) analysis of purity-corrected significant correlations between ARPs expression level and tumor-infiltrating immune cells in GBM. (**A**) Correlation analysis of BAK1 expression level with CD8+ T cell; (**B**) Correlation analysis of CDKN1B expression level with CD8+ T cell, and (**C**) Macrophage; (**D**) Correlation analysis of CHMP6 expression level with CD8+ T cell; (**E**) Correlation analysis of EFGR expression level with CD8+ T cell; (**F**) Correlation analysis of GAPDH expression level with CD8+ T cell; (**G**) Correlation analysis of NDUFB9 expression level with CD8+ T cell; (**H**) Correlation analysis of SUPT3H expression level with CD8+ T cell

Autophagy has been shown to play a crucial role in immune modulation, such as endogenous and exogenous antigens fusing with MHC I and MHC II for antigen presentation and recognition by TCRs on the surface of T cells [22]. The tumor immune microenvironment consisted of massive immune cell subsets surrounding cancer cells, including B cells, CD4 + T cells, CD8 + T cells, neutrophils, macrophages, and dendritic cells. We therefore hypothesized that immunity in GBM could be linked to the seven signature genes expression. We found that cases with high expression of *NDUFB9, GAPDH, CDKN1B, CHMP6*, and *EGFR* showed significantly increased infiltration of CD8 + T cells. On the other hand, the expression of *BAK1*, and *SUPT3H* expression was not correlated with CD8 + cells infiltration. CDKN1B expression was also positively correlated with increased Macrophage level (Fig. 5).

### Correlation between signature genes expression and cytokine receptor in the tumour microenvironment of GBM

**Fig. 6:**
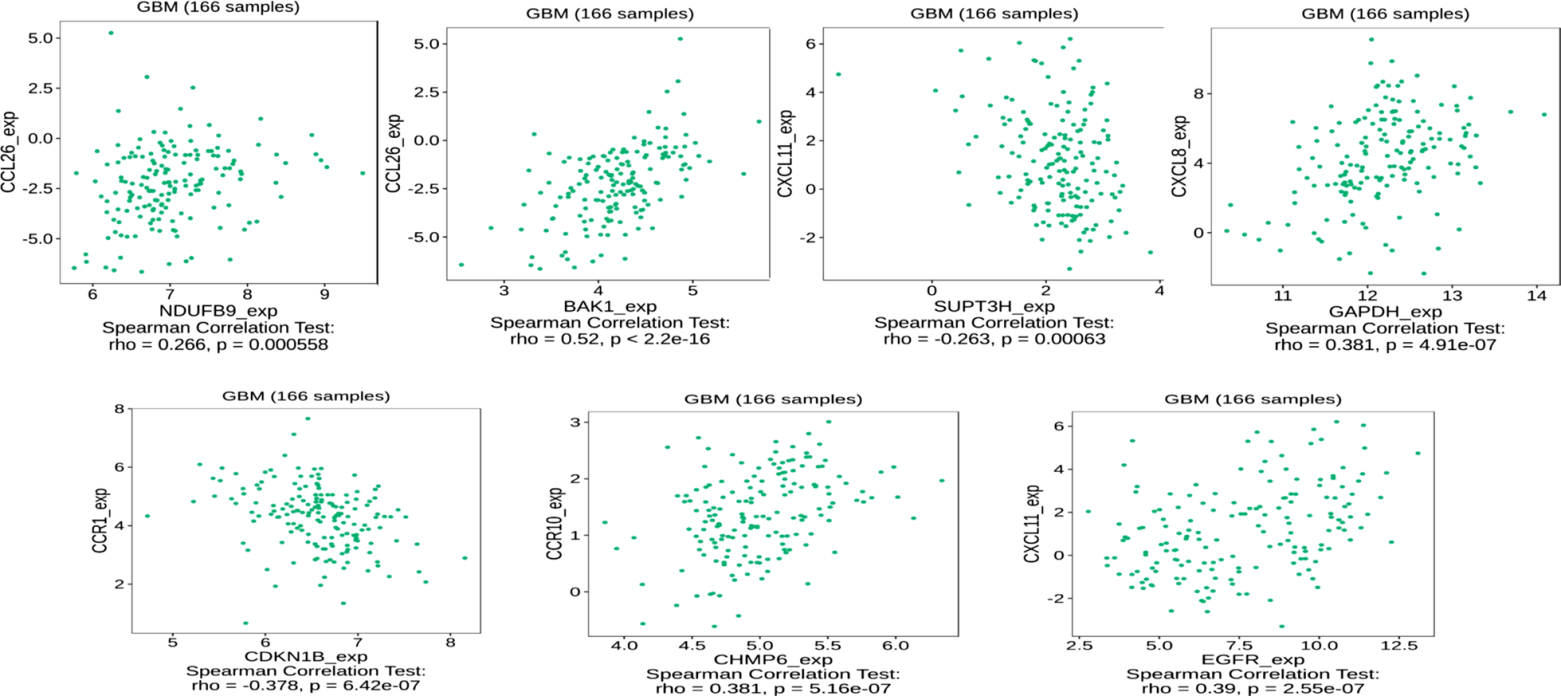
Spearman’s correlation of ARPs with cytokine receptor in tumor immune microenvironment

In the tumor microenvironment, the crosstalk between tumor cells and cytokines in the tumor microenvironment is essential for tumorigenesis. Hence, we further investigated the relationship between the signature genes and the expression of cytokine receptor expression [32]. Our results showed strong positive correlations between signature genes and C–C motif chemokine ligand (CCL), C–C chemokine receptor (CCR), and CXC chemokine family, which suggests that the hallmark of the tumor microenvironment, the chemokine, are involved in crosstalk with autophagy-signature genes and might have an intersection with autophagy pathways and affect the prognosis in GBM patients (figures with the highest correlation for each gene and cytokine receptor were exhibited). Interestingly, the *BAK1* and CCL26 have the highest correlation of 0.52 (p < 2.2e-16), which suggests autophagy genes were significantly influenced by the expression of cytokines receptor in TME in GBM (Fig. 6).

### Drug sensitivity analysis of signature genes

**Fig. 7:**
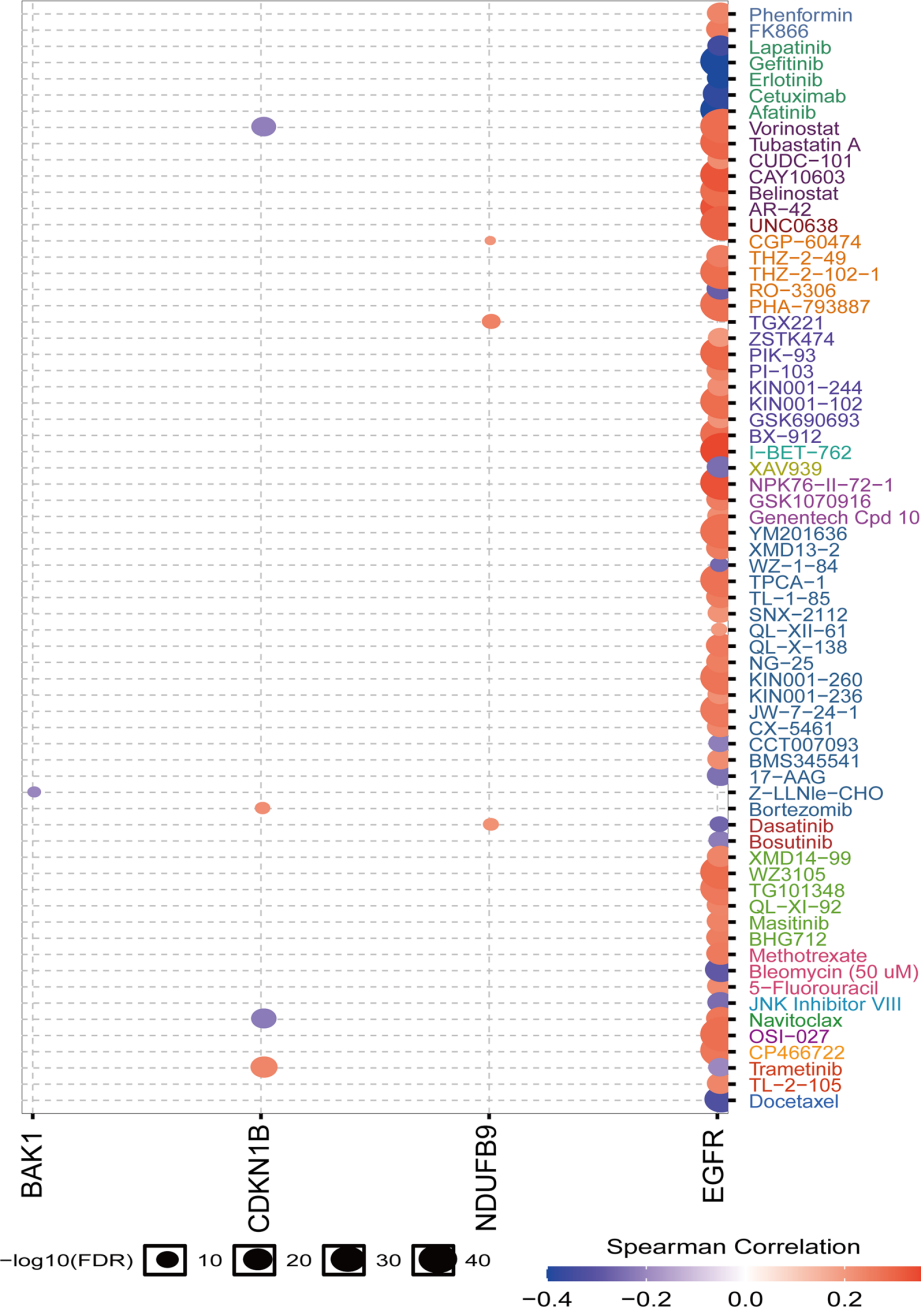
Drug sensitivity analysis of ARPs in GBM based on the GDSC drug sensitivity database. The spearman correlation represents the correlation of gene expression with the small molecular drug. A positive correlation indicates that high gene expression is resistant to the drug and vice versa

Current targeted therapy has a limited effect on GBM, and could easily induce drug resistance [33]. Hence, in addition to explore the correlation between signature genes expression and immune microenvironment, we also investigated the effect of signature genes on the drug sensitivity of GBM patients. Using the GDSC database, we identified whether patients with dysregulation of autophagy-signature genes have potential selective small molecular drugs. The results showed that high expression of *EGFR* was associated with sensitivity to some of the small molecular drugs (Lapatinib, Gefitinib, Erlotinib, Cetuximab, Afatinib, et al.); while high expression of CDKN1B was sensitivity to Vorinostat; high expression of BAK1 was sensitivity to Z-LLNle-CHO (Fig. 7), which further support the autophagy-signature for the potential optimization of targeted therapy in patients with GBM. Overall, our data provide a basis for further elucidation of drug sensitivity prediction through the autophagy signature.

## Discussion

Autophagy plays a controversial role in cancer development and has been widely discussed during the past decade [34, 35]. Some of the autophagy-related genes have been shown to be promising treatment targets [14, 36, 37]. However, the expression of these autophagy genes in stratifying the prognostic risk in GBM patients is still unclear. Therefore, the development of a meaningful autophagy signature may provide clinicians with a selection of the high-risk population.

IIn our study, we first examined different expressed patterns of autophagy genes in GBM tissues and normal brain tissues, and then profiled a 7-gene autophagy-related signature that could predict the prognosis of GBM, which was further validated by an external independent data set. Moreover, we showed that seven signature genes are potential drug-sensitive genes and prognosis markers, which were linked to tumor immune cell infiltration, and cytokine-cytokine receptor.in TME.

To our knowledge, this is the first study to simultaneously measure correlations between ARPs, immune cell infiltration, and cytokine receptors in the tumor microenvironment using digital computational analysis and construct an autophagy-related prognostic model for GBM. These results indicate that autophagy-related genes might be potential therapeutic targets in GBM.

To study the potential mechanism of prognosis difference between high-risk and low-risk GBM groups, we drafted the genetic profile of the seven signature genes, which is in accordance with the large-scale genomic analysis result of human cancers that indicates the loss or mutation of core autophagy genes is uncommon [38]. Autophagy impacts cellular metabolism, the proteome, and organelle numbers and quality, which alter cell functions in diverse ways [39]. Hence, dysfunctional autophagy contributes to cancer predominantly via impacting cellular metabolism, immune, and tumor microenvironment [38, 40, 41]. Camuzard O, et al. reported a key role that autophagy in the crosstalk between tumor and microenvironment in various of cancers [42]. Additionally, Jun Wei et al. unveiled the interplay between autophagy and metabolic programming as a new mechanism to enforce tumor microenvironment immunity function, which could act as strategies that block autophagy in tumor cells for added benefits in cancer therapy [43]. Consistent with our data, these findings further support an important impact of autophagy as potential therapeutic target for GBM patients. Our study of the autophagy-signature can help to understand the activation of autophagy in GBM patients may improve therapeutic approaches with differing molecule mechanisms.

We further analyzed the dysregulated signaling pathways between high- and low-risk groups affected by autophagy. As described in GO and KEGG analyses, the results revealed that all differential genes between high- and low-risk groups were significantly enriched in terms of cancer proliferation (negative regulation of mitotic cell cycle), metabolism (HIF-1 signaling pathway), and immune microenvironment (ErbB signaling pathway, and MicroRNAs in cancer) related pathways. Cancer cell proliferation, abnormality of metabolism, and reshaped tumor microenvironment are hallmarks of tumor growth and metastasis [44]. Thus, we might suppose that the 7-risk signature genes played a key role in contributing to the poor prognosis of GBM probably by regulating cancer cell metabolism, immune, or tumor microenvironment factors.

Another important discovery of our research is that autophagy signature genes are linked to the infiltration level of CD4 + , and CD8 + T cells in GBM. Given that CD4 + T cells, CD8 + T cells and immune suppression by *CD4* + *FOXP3* + regulatory T (Treg) cells represent two major factors impacting response to cancer immunotherapy [45], this finding suggests that the dysregulated autophagy pathways may affect the response of immunotherapy. But their precise integration into the autophagy and immune microenvironment of human cancer has not been established.

This discovery prompted us to examine the potential underlying mechanisms that autophagy processes contribute to the tumor microenvironment, such as chemokines and cytokine receptors. Chemokines are produced by and target tumor and tumor-associated host cells, they form a large intercellular signaling network through interacting by a large number of ligand-selective surface receptors [46]. We indeed found that a group of cytokine receptors presented correlations with autophagy signature genes. Of note, there seems to be an up to 50% likelihood of positive correlationship between *BAK1* and CCL; 37.8% negative correlationship between *CDKN1B* and CCR; 39% positive correlationship between *EGFR* and CXCL. Chemokines, a large family of cytokines with chemotactic activity, and their cognate receptors are expressed by both cancer and stromal cells [46]. Previous studies have shown that tumor-associated macrophages (TAMs) widely express CXCR1/2 and be recruited to the tumor bed through the CXCL8-CXCR1/2 axis facilitating immune escape [47]. Consistently, Murakami T et al. found that the loss of tumor endogenous chemokines and receptors is a common mechanism of immune escape [48]. Besides, Seema Singh et al. reveal that CXCR1 and CXCR2 may be a future therapeutic interventions for the modulating of cellular phenotypes associated with melanoma tumor growth and angiogenesis through knock-down of CXCR1 or CXCR2 [49]. Out data indicate that autophagy risk factors are closely related to cytokine receptors in the microenvironment, which may regulate the expression of cytokine receptors in the microenvironment, leading to immune escape, and affect the prognosis of GBM patients.

The advantage of our study is that we performed a systematic analysis of microarray data and RNAseq data, which suggested the involvement of autophagy-related signature in prognosis, drug sensitivity, and microenvironment immune cell infiltration in GBM. It does have some limitations. First, the data come entirely from open databases and have not been verified experimentally. Second, The expression of autophagy signature has a certain correlation with drug sensitivity, tumor microenvironment, tumor immunity, but there is a lack of detailed experimental verification.

## Conclusion

In summary, we developed an autophagy-related gene expression model in GBM that could independently predict the overall survival of GBM patients. Furthermore, further investigations into the molecular mechanisms of autophagy reveal that the expression of autophagy signature genes were also related to tumor drug sensitivity, tumor microenvironment, and immune cell infiltration. Of note, our results suggest that the use of a targeting autophagy therapy might be a promising future strategy for treating GBM. Thus, our 7-gene autophagy-related signature may predict the survival risk of GBM patients, and also guide the therapeutic approaches used for those patients.

## Supplementary Information

**Additional file 1.** ARGs selected in this study.

**Additional file 2.** Univariate Cox regression analysis detected a total of 30 ARGs, which were related to the GBM prognosis.

**Additional file 3.** The LASSO-regression analysis identified 19 genes with non-zero coefficients.

**Additional file 4.** All the data sets analyzed in this research.

**Additional file 5: Fig. S1**: Principle components analysis of autophagy-related genes in GBM and normal brain samples.

## Data Availability

Level 3 RNA-seq data and corresponding clinical data for xx GBM were acquired from the data portal for TCGA (https://portal.gdc.cancer.gov/), using R/Bioconductor package TCGAbiolinks, which are publicly available (https://portal.gdc.cancer.gov); microarray datasets (accession number: GSE4290, GSE7696, GSE50161, and GSE7696) for this study are openly available in Gene Expression Omnibus database.

## References

[1] Ostrom QT, Gittleman H, Truitt G, Boscia A, Kruchko C, Barnholtz-Sloan JS (2018). CBTRUS statistical report: primary brain and other central nervous system tumors diagnosed in the United States in 2011–2015. Neuro Oncol..

[2] Shergalis A, Bankhead A, Luesakul U, Muangsin N, Neamati N (2018). Current challenges and opportunities in treating glioblastoma. Pharmacol Rev.

[3] Szopa W, Burley TA, Kramer-Marek G, Kaspera W (2017). Diagnostic and therapeutic biomarkers in glioblastoma: current status and future perspectives. Biomed Res Int..

[4] Burger PC, Minn AY, Smith JS (2001). Losses of chromosomal arms 1p and 19q in the diagnosis of oligodendrogliomas. A study of paraffin-embedded sections. Mod Pathol..

[5] Labussière M, Idbaih A, Wang XW (2010). All the 1p19q codeleted gliomas are mutated on IDH1 or IDH2. Neurology.

[6] Esteller M, Garcia-Foncillas J, Andion E (2000). Inactivation of the DNA-repair gene MGMT and the clinical response of gliomas to alkylating agents. N Engl J Med.

[7] Chung C, Seo W, Silwal P, Jo EK (2020). Crosstalks between inflammasome and autophagy in cancer. J Hematol Oncol.

[8] Liu T (2019). Regulation of Inflammasome by Autophagy. Adv Exp Med Biol.

[9] Ding Y, Li T, Li M, Tayier T, Zhang M, Chen L, Feng S (2021). A Novel Autophagy-Related lncRNA Gene Signature to Improve the Prognosis of Patients with Melanoma. Biomed Res Int.

[10] Meng D, Jin H, Zhang X, Yan W, Xia Q, Shen S, Xie S, Cui M, Ding B, Gu Y, Wang S (2021). Identification of autophagy-related risk signatures for the prognosis, diagnosis, and targeted therapy in cervical cancer. Cancer Cell Int.

[11] Zhao D, Sun X, Long S, Yao S (2021). An autophagy-related long non-coding RNA signature for patients with colorectal cancer. Physiol Int..

[12] Mizushima N, Levine B, Cuervo AM, Klionsky DJ (2008). Autophagy fights disease through cellular self-digestion. Nature.

[13] Doria A, Gatto M, Punzi L (2013). Autophagy in human health and disease. N Engl J Med.

[14] Vehlow A, Cordes N (2019). DDR1 (discoidin domain receptor tyrosine kinase 1) drives glioblastoma therapy resistance by modulating autophagy. Autophagy.

[15] Parisi S, Corsa P, Raguso A (2015). Temozolomide and radiotherapy versus radiotherapy alone in high grade gliomas: a very long term comparative study and literature review. Biomed Res Int..

[16] Moussay E, Kaoma T, Baginska J, Muller A, Van Moer K, Nicot N, Nazarov PV, Vallar L, Chouaib S, Berchem G, Janji B (2011). The acquisition of resistance to TNFα in breast cancer cells is associated with constitutive activation of autophagy as revealed by a transcriptome analysis using a custom microarray. Autophagy.

[17] Subramanian A, Tamayo P, Mootha VK (2005). Gene set enrichment analysis: a knowledge-based approach for interpreting genome-wide expression profiles. Proc Natl Acad Sci U S A.

[18] Song K, Li L, Zhang G (2017). Bias and correction in RNA-seq data for marine species. Mar Biotechnol (NY).

[19] Sun L, Hui AM, Su Q, Vortmeyer A, Kotliarov Y, Pastorino S, Passaniti A, Menon J, Walling J, Bailey R, Rosenblum M, Mikkelsen T, Fine HA (2006). Neuronal and glioma-derived stem cell factor induces angiogenesis within the brain. Cancer Cell.

[20] Lambiv WL, Vassallo I, Delorenzi M, Shay T, Diserens AC, Misra A, Feuerstein B, Murat A, Migliavacca E, Hamou MF, Sciuscio D, Burger R, Domany E, Stupp R, Hegi ME (2011). The Wnt inhibitory factor 1 (WIF1) is targeted in glioblastoma and has a tumor suppressing function potentially by induction of senescence. Neuro Oncol.

[21] Griesinger AM, Birks DK, Donson AM, Amani V, Hoffman LM, Waziri A, Wang M, Handler MH, Foreman NK (2013). Characterization of distinct immunophenotypes across pediatric brain tumor types. J Immunol.

[22] Li YY, Feun LG, Thongkum A, Tu CH, Chen SM, Wangpaichitr M, Wu C, Kuo MT, Savaraj N (2017). Autophagic mechanism in anti-cancer immunity: its pros and cons for cancer therapy. Int J Mol Sci.

[23] Irizarry RA, Hobbs B, Collin F (2003). Exploration, normalization, and summaries of high density oligonucleotide array probe level data. Biostatistics.

[24] Cerami E, Gao J, Dogrusoz U, Gross BE, Sumer SO, Aksoy BA, Jacobsen A, Byrne CJ, Heuer ML, Larsson E, Antipin Y, Reva B, Goldberg AP, Sander C, Schultz N (2012). The cBio cancer genomics portal: an open platform for exploring multidimensional cancer genomics data. Cancer Discov.

[25] Johnson WE, Li C, Rabinovic A (2007). Adjusting batch effects in microarray expression data using empirical Bayes methods. Biostatistics.

[26] Kawaguchi A, Iwadate Y, Komohara Y, Sano M, Kajiwara K, Yajima N, Tsuchiya N, Homma J, Aoki H, Kobayashi T, Sakai Y, Hondoh H, Fujii Y, Kakuma T, Yamanaka R (2012). Gene expression signature-based prognostic risk score in patients with primary central nervous system lymphoma. Clin Cancer Res.

[27] da Huang W, Sherman BT, Lempicki RA (2009). Systematic and integrative analysis of large gene lists using DAVID bioinformatics resources. Nat Protoc.

[28] Ru B, Wong CN, Tong Y, Zhong JY, Zhong SSW, Wu WC, Chu KC, Wong CY, Lau CY, Chen I, Chan NW, Zhang J (2019). TISIDB: an integrated repository portal for tumor-immune system interactions. Bioinformatics.

[29] Li B, Li T, Liu JS, Liu XS (2020). Computational deconvolution of tumor-infiltrating immune components with bulk tumor gene expression data. Methods Mol Biol.

[30] Li T, Fan J, Wang B, Traugh N, Chen Q, Liu JS, Li B, Liu XS (2017). TIMER: a web server for comprehensive analysis of tumor-infiltrating immune cells. Cancer Res.

[31] Yang W, Soares J, Greninger P, Edelman EJ, Lightfoot H, Forbes S, Bindal N, Beare D, Smith JA, Thompson IR, Ramaswamy S, Futreal PA, Haber DA, Stratton MR, Benes C, McDermott U, Garnett MJ (2013). Genomics of drug sensitivity in cancer (GDSC): a resource for therapeutic biomarker discovery in cancer cells. Nucleic Acids Res..

[32] Weiss T, Puca E, Silginer M, Hemmerle T, Pazahr S, Bink A, Weller M, Neri D, Roth P (2020). Immunocytokines are a promising immunotherapeutic approach against glioblastoma. Sci Transl Med..

[33] Jackson CM, Choi J, Lim M (2019). Mechanisms of immunotherapy resistance: lessons from glioblastoma. Nat Immunol.

[34] White E (2012). Deconvoluting the context-dependent role for autophagy in cancer. Nat Rev Cancer.

[35] Orvedahl A, McAllaster MR, Sansone A (2019). Autophagy genes in myeloid cells counteract IFNγ-induced TNF-mediated cell death and fatal TNF-induced shock. Proc Natl Acad Sci USA.

[36] Zhou Y, Wang Y, Zhou W (2019). YAP promotes multi-drug resistance and inhibits autophagy-related cell death in hepatocellular carcinoma via the RAC1-ROS-mTOR pathway. Cancer Cell Int..

[37] Liao X, Fan Y, Hou J (2019). Identification of chaetocin as a potent non-ROS-mediated anticancer drug candidate for gastric cancer. J Cancer.

[38] Tome-Garcia J, Erfani P, Nudelman G (2018). Analysis of chromatin accessibility uncovers TEAD1 as a regulator of migration in human glioblastoma. Nat Commun.

[39] Kim KH, Lee MS (2014). Autophagy–a key player in cellular and body metabolism. Nat Rev Endocrinol.

[40] Ding T, Ma Y, Li W (2011). Role of aquaporin-4 in the regulation of migration and invasion of human glioma cells. Int J Oncol.

[41] Chen Y, Gao F, Jiang R (2017). Down-regulation of AQP4 expression via p38 MAPK signaling in temozolomide-induced glioma cells growth inhibition and invasion impairment. J Cell Biochem.

[42] Camuzard O, Santucci-Darmanin S, Carle GF, Pierrefite-Carle V (2020). Autophagy in the crosstalk between tumor and microenvironment. Cancer Lett.

[43] Wei J, Long L, Yang K, Guy C, Shrestha S, Chen Z, Wu C, Vogel P, Neale G, Green DR, Chi H (2016). Autophagy enforces functional integrity of regulatory T cells by coupling environmental cues and metabolic homeostasis. Nat Immunol.

[44] Hanahan D, Weinberg RA (2011). Hallmarks of cancer: the next generation. Cell.

[45] Chao JL, Korzinkin M, Zhavoronkov A, Ozerov IV, Walker MT, Higgins K, Lingen MW, Izumchenko E, Savage PA (2021). Effector T cell responses unleashed by regulatory T cell ablation exacerbate oral squamous cell carcinoma. Cell Rep Med.

[46] Mollica Poeta V, Massara M, Capucetti A, Bonecchi R (2019). Chemokines and chemokine receptors: new targets for cancer immunotherapy. Front Immunol.

[47] Li BH, Garstka MA, Li ZF (2020). Chemokines and their receptors promoting the recruitment of myeloid-derived suppressor cells into the tumor. Mol Immunol.

[48] Murakami T, Cardones AR, Finkelstein SE, Restifo NP, Klaunberg BA, Nestle FO, Castillo SS, Dennis PA, Hwang ST (2003). Immune evasion by murine melanoma mediated through CC chemokine receptor-10. J Exp Med.

[49] Singh S, Sadanandam A, Nannuru KC, Varney ML, Mayer-Ezell R, Bond R, Singh RK (2009). Small-molecule antagonists for CXCR2 and CXCR1 inhibit human melanoma growth by decreasing tumor cell proliferation, survival, and angiogenesis. Clin Cancer Res.

