# Supplementary material for: Identification of a novel autophagy-related prognostic signature and small molecule drugs for glioblastoma by bioinformatics
Source: BMC Med Genomics. 2022 May 12;15:111. doi: 10.1186/s12920-022-01261-5 (PMC9097333; doi:10.1186/s12920-022-01261-5)
Supplement: Supplementary file 2 — Additional file 2. Univariate Cox regression analysis detected a total of 30 ARGs, which were related to the GBM prognosis. [file 12920_2022_1261_MOESM2_ESM.docx]

| id | HR | HR.95L | HR.95H | *p*-value |
| --- | --- | --- | --- | --- |
| ITGA6 | 1.801733801 | 1.28292552 | 2.530345401 | 0.000679205 |
| MRPS2 | 0.285175062 | 0.132097048 | 0.615644459 | 0.001396369 |
| BAK1 | 0.091031131 | 0.019380322 | 0.42758148 | 0.002394111 |
| PDK4 | 0.573258938 | 0.392756107 | 0.83671725 | 0.003927314 |
| SAR1A | 0.228883077 | 0.079457401 | 0.659315079 | 0.006301912 |
| DZANK1 | 3.639334528 | 1.36054607 | 9.734882262 | 0.010074112 |
| NRBF2 | 0.512150887 | 0.306283482 | 0.856391372 | 0.010741886 |
| CHMP6 | 0.299727688 | 0.114257687 | 0.786263835 | 0.014339231 |
| ERBB2 | 3.842911526 | 1.285072381 | 11.49193556 | 0.016008003 |
| PI4K2A | 0.176931413 | 0.042623452 | 0.73444838 | 0.017081197 |
| SUPT3H | 0.369142477 | 0.159380405 | 0.854974414 | 0.020039845 |
| SNF8 | 0.430985204 | 0.211764443 | 0.877145583 | 0.020259163 |
| VPS33B | 0.346099909 | 0.139443283 | 0.859024149 | 0.022161547 |
| RAB23 | 1.754896131 | 1.078547114 | 2.855378676 | 0.023548949 |
| EGFR | 1.413721198 | 1.045161234 | 1.912248141 | 0.024666815 |
| LIX1 | 1.286593371 | 1.032477941 | 1.603252173 | 0.02478988 |
| ITGB4 | 3.143006337 | 1.155157809 | 8.551635769 | 0.024935728 |
| NDUFB9 | 0.431918152 | 0.206801647 | 0.90208803 | 0.025470572 |
| MYC | 0.715875641 | 0.533581125 | 0.960449892 | 0.025808719 |
| ANXA5 | 1.586757819 | 1.047294396 | 2.404099922 | 0.029409643 |
| KRCC1 | 2.076383213 | 1.050626832 | 4.103614258 | 0.035548649 |
| CDKN1B | 1.562084675 | 1.026523383 | 2.377060836 | 0.037326941 |
| PELP1 | 0.361789157 | 0.138709231 | 0.943638672 | 0.037657283 |
| COX8A | 0.412035808 | 0.177915735 | 0.95423548 | 0.038518645 |
| GAPDH | 1.979372872 | 1.031096314 | 3.799758486 | 0.040170071 |
| MYOM1 | 2.050826027 | 1.024255289 | 4.106288186 | 0.042599031 |
| TM9SF1 | 0.476098486 | 0.231720327 | 0.978204074 | 0.043389134 |
| RAB24 | 0.471434749 | 0.226892951 | 0.979540006 | 0.043866151 |
| TEAD4 | 0.525075895 | 0.279687707 | 0.985759074 | 0.045006251 |
| TBC1D5 | 3.050979981 | 1.02139197 | 9.113522643 | 0.045730269 |

**Additional file 2** Univariate analysis identified autophagy genes with a *p* value less than 0.05.
