# Supplementary material for: Identification of a novel autophagy-related prognostic signature and small molecule drugs for glioblastoma by bioinformatics
Source: BMC Med Genomics. 2022 May 12;15:111. doi: 10.1186/s12920-022-01261-5 (PMC9097333; doi:10.1186/s12920-022-01261-5)
Supplement: Supplementary file 4 — Additional file 4. All the data sets analyzed in this research. [file 12920_2022_1261_MOESM4_ESM.doc]

| **PCA** | **42** | **23** | **GSE4290** | **non-tumor** | **Normal (42)** |
| --- | --- | --- | --- | --- | --- |
|  |  | **2** | **GSE15824** | **tissue: brain** |  |
|  |  | **4** | **GSE7696** | **disease status: non-tumoral** |  |
|  |  | **13** | **GSE50161** | **normal brain** |  |
|  | **197** | **81** | **GSE4290** | **glioblastoma, grade 4** | **Tumor (207)** |
|  |  | **12** | **GSE15824** | **tissue: brain** |  |
|  |  | **80** | **GSE7696** | **disease status: GBM** |  |
|  |  | **34** | **GSE50161** | **tissue: glioblastoma** |  |
| **discovery cohort** | **156** | **156** | **TCGA** | **156** | **Tumor** |
| **validation data set** | **80** | **80** | **GSE7696** | **80** | **Tumor** |

Additional file 4: List of data sets in this article
