# Supplementary figures and images for: Identification of a novel autophagy-related prognostic signature and small molecule drugs for glioblastoma by bioinformatics
Source: BMC Med Genomics. 2022 May 12;15:111. doi: 10.1186/s12920-022-01261-5 (PMC9097333; doi:10.1186/s12920-022-01261-5)

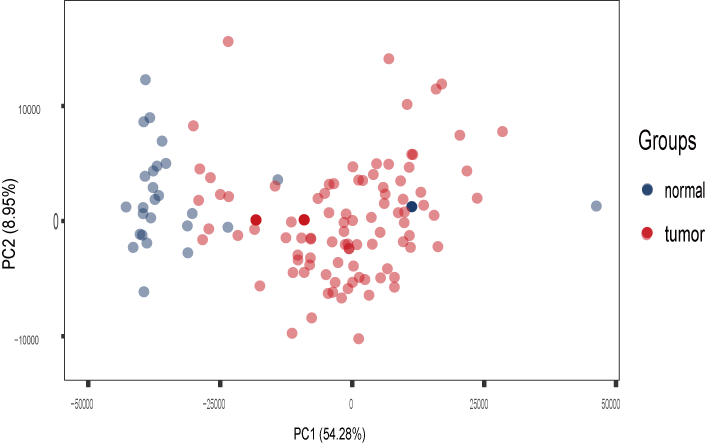

Supplement: Supplementary file 5 — Additional file 5: Fig. S1: Principle components analysis of autophagy-related genes in GBM and normal brain samples. [file 12920_2022_1261_MOESM5_ESM.tif]
